# Deciphering the pathogenesis of tendinopathy: a three-stages process

**DOI:** 10.1186/1758-2555-2-30

**Published:** 2010-12-13

**Authors:** Sai-Chuen Fu, Christer Rolf, Yau-Chuk Cheuk, Pauline PY Lui, Kai-Ming Chan

**Affiliations:** 1 Department of Orthopaedics & Traumatology, Faculty of Medicine, The Chinese University of Hong Kong, Prince of Wales Hospital, Hong Kong Special Administrative Region, PR China; 2 The Hong Kong Jockey Club Sports Medicine and Health Sciences Centre, Faculty of Medicine, The Chinese University of Hong Kong, Prince of Wales Hospital, Hong Kong Special Administrative Region, PR China; 3 Department of Orthopaedic Surgery, Huddinge University Hospital, CLINTEC, Karolinska Institutet, Stockholm, Sweden

## Abstract

Our understanding of the pathogenesis of "tendinopathy" is based on fragmented evidences like pieces of a jigsaw puzzle. We propose a "failed healing theory" to knit these fragments together, which can explain previous observations. We also propose that albeit "overuse injury" and other insidious "micro trauma" may well be primary triggers of the process, "tendinopathy" is not an "overuse injury" per se. The typical clinical, histological and biochemical presentation relates to a localized chronic pain condition which may lead to tendon rupture, the latter attributed to mechanical weakness. Characterization of pathological "tendinotic" tissues revealed coexistence of collagenolytic injuries and an active healing process, focal hypervascularity and tissue metaplasia. These observations suggest a failed healing process as response to a triggering injury. The pathogenesis of tendinopathy can be described as a three stage process: injury, failed healing and clinical presentation. It is likely that some of these "initial injuries" heal well and we speculate that predisposing intrinsic or extrinsic factors may be involved. The injury stage involves a progressive collagenolytic tendon injury. The failed healing stage mainly refers to prolonged activation and failed resolution of the normal healing process. Finally, the matrix disturbances, increased focal vascularity and abnormal cytokine profiles contribute to the clinical presentations of chronic tendon pain or rupture. With this integrative pathogenesis theory, we can relate the known manifestations of tendinopathy and point to the "missing links". This model may guide future research on tendinopathy, until we could ultimately decipher the complete pathogenesis process and provide better treatments.

## Introduction

In the past decades, our concepts on chronic tendon pain have evolved from "tendinitis" which focused on clinical inflammatory signs, into "tendinosis" which stressed the pathologic features of the free tendon as observed by histology and biochemistry, and then "tendinopathy" which declared nothing further about its nature, just introducing a new label for chronic tendon and insertion problems in general [1]. Woo and Renstrom [2] concludes that the pathogenesis, etiology and mechanisms in most of the myriads of conditions related to tendinopathy are unknown. However, with clear definitions outlining and discriminating the various diagnoses of "tendinopathy", it is still possible to propose a unified model for the pathogenesis based on available experimental evidences, which we propose as our theory to be proved or rejected by future investigation.

In general, tendinopathy is characterized by longstanding localized activity-related pain and the patients in general respond poorly to most "conservative treatments". However, a wide spectrum of tendon pathologies is put under the umbrella entity of tendinopathy based on some common features [3] (Table 1), leading to an impression that there is no single general pathogenesis or aetiology involved which can explain all conditions. If so, we firmly believe that these pathologies should be classified as different entities. As the current research evidences are confusing, it is very important to identify if there are common denominators and diversifiers for various manifestations of what we loosely call "tendinopathy" to help us understand the pathogeneses of these conditions.

**Table 1:** Involvement of failed healing in different manifestations of tendinopathies.

Injury	Healing responses	Failed Healing	Histopathological changes	Clinical presentation	Different manifestations
• Overuse • Previous traumatic injury • Xenobiotics • Pathogens	Inflammation	⇒	Sustained pro-inflammatory cytokines	• Pain • Mechanical weakness	• "Tendinitis" • Paratendinitis • Insertional tendinopathy • Overuse tendon injuries • Spontaneous rupture • Activity-related pain • Calcified tendinopathy
			
	Neovascularization	⇒	Hypervascularity		
			
	Innervation	⇒	Increased neuropeptides and innervation		
			
	Cell recruitment/apoptosis	⇒	Hypercellularity, increased apoptosis		
			
	Matrix synthesis	⇒	Mucoid, lipoid, calcific degeneration		
				
	Tenogenic differentiation/apoptosis	⇒			
			
	Matrix remodeling	⇒	Collagenolysis, tendon adhesion		

In this article, we review previous investigations according to the nature of the studies, for example, clinical observations, characterization of clinical samples, evaluation of treatments to patients with tendinopathy, animal models of tendinopathy and cell culture studies related to the effects of risk factors. Certainly we do not answer all questions with the integration of various research evidences, but we take the bold step to propose an integrative theory for the pathogenesis of tendinopathy based on the underlying messages in these studies.

## Clinical observations of tendinopathy

To understand the pathogenesis of what today is labeled as "tendinopathy" we have to make some clear distinctions. Firstly we must consider the varying clinical presentations. Most tendon problems are presented to the clinician either as a rupture or localized pain, often including stiffness and swelling. Symptomatic tendinopathy refers to chronic localized pain with "degenerative" changes in tendons as observed by imaging or histology; while asymptomatic tendinopathy is identified from ruptures or partial rupture cases shown to be associated with non symptomatic pre-existing degenerative changes. Pathologies primarily manifested as passive loss of range of motion (i.e. trigger finger, frozen shoulder, etc) are not considered as tendinopathy in this discussion. Dating back to 1938, Codman reported degeneration in complete ruptures of rotator cuff [4]. Kannus and Josza reported in 1991 from a large number of histological samples that an absolute majority of patients with complete Achilles tendon ruptures had pathologic alterations which he described as "mucoid degeneration" [5]. This "mucoid degeneration" is almost equivalent to the histological alterations characterized for tendinosis [6]. It is very likely that these pathological changes in tendons imposed mechanical weakness and higher susceptibility to ruptures. Similar histopathological characteristics were also described in clinical samples of symptomatic tendinopathy [7,8]. It suggests that the "typical" histopathological changes characterized by tendon degeneration may not necessarily be directly linked to increased nociception giving the patients warning signals; while in painful cases, the mechanically weaker tendons may be protected from ruptures due to decreased impact levels since painful activities will be avoided.

Secondly, we must consider the etiology and epidemiology. Unfortunately, well defined epidemiological studies on "tendinopathy" are virtually nonexistent. Age-related changes in tendons were reported [9], but tendinopathy is not an age-related degeneration because similar pathological changes are observed in young people [10]. Higher number of cases in males presented in clinical studies [11,12] may not reflect higher susceptibility of male gender to tendinopathy; on the contrary, it is reported that female gender was more susceptible to repetitive trauma in rotator cuff [13] and female cyclists suffer a higher risk for "overuse injury" in general than their male counterparts [14]. There were significant gender differences in tendon microcirculation [15] and the neuropeptide responsiveness in rabbit tendon explants was influenced by gender and pregnancy [16]. Diabetes [17] and metabolic alterations such as dislipidemia [18] has been proposed as risk factor for developing tendinopathy. These findings suggest that the hormonal background may affect the development of tendinopathy. Fluoroquinolone [19] and corticosteroids [20] were found to be associated with Achilles tendon ruptures; suggesting pharmacological influence on the development of tendon pathology. Overuse, repetitive strain or mechanical overload to tendons are considered as primary trigger of symptomatic tendinopathy in various regions [21], as implied by the names such as "jumper's knee", "runner's heel", "swimmer's shoulder" and "tennis elbow". The prevalence of supraspinatous tendinopathy could be as high 69 % in elite swimmers [22]. However, there are frequent tendinopathy cases (pain or rupture) in the non-athlete population [23,20]. Thus overuse injury should not be equated to tendinopathy, but it may be one of the major triggers of the pathological development in some individuals. Furthermore, overuse as a risk factor for tendinopathy is not simply a quantitative increase in activities, but may also be attributed to improper gait or training errors [24,25].

Thirdly, the anatomical sites of tendinopathic changes add further complexity. Since overuse or cumulative trauma may also affect other peritendinous tissues, tendinopathy was sometimes presented with pathological changes in tenosynovium, bursa and nerves. Our discussion on the pathogenesis of tendinopathy should be focused on changes primarily initiated and observed in tendons; otherwise the pathogenesis pathways will be very heterogeneous. It follows that infectious tenosynovitis, bursitis, adhesive capsulitis or tendon and nerve entrapment in case of carpel tunnel syndrome will not be included in our model, but "paratenonitis" [26] and "insertional tendinopathies" [27] will be discussed since they are parts of a tendon. The pathological changes in different forms of tendinopathy are localized in different regions of the affected tendons, for example, the proximal deep posterior portion of the patellar tendon is affected in patellar tendinopathy, while mid-substance or insertion pathological changes can be observed in Achilles tendinopathy. The medial musculotendinous junction or lateral Humerus insertion was affected in Rotator cuff tendinopathy, while in lateral epicondylitis the fascial collagen structure on the extensor carpi radialis brevis tendon was pathological. Based on the involvement of pathological changes in the paratenon, different sub-classes can be further identified in Achilles tendinopathy [28]. Owing to these variations in the sites of pathological changes, it suggests the common denominator of the pathogenesis of tendinopathy may probably involve a process that can affect all parts of tendons [29], including musculotendinous junction, mid-substance, insertion and paratenons. The "communication" between these structures around the tendons is poorly investigated.

## Medical imaging of tendinopathy

Tendinopathy exhibited characteristic pathological changes which are visible under ultrasound or magnetic resonance imaging (MRI). Tendon thickening or swelling is revealed, localized hypoechogenic signals were detected by ultrasound [30] and an increased T1 and T2 contrast signal was shown by MRI [31,32]. It suggests an increased water content which is probably related to increased accumulation of water-retaining proteoglycans. Doppler ultrasound imaging showed increased vascularity and blood flow in the pathological regions but the oxygen tension was not significantly different [33]. These findings suggested an inflammatory component [34] with localized changes in the tendinous matrix and hypervascularity may be associated with the pathogenesis of tendinopathy.

## Characterization of clinical samples of tendinopathy

Direct investigation of tendinopathy started with histological examination of the pathological tissues. Classical characteristics of "tendinosis" include degenerative changes in the collagenous matrix, hypercellularity, hypervascularity and a lack of inflammatory cells which has challenged the original misnomer "tendinitis" [35,6]. Further characterizations are basically extrapolation of these findings, for examples, measurement of proliferation and apoptosis to explain the changes in cellularity [36,37], detection of extracellular matrix components [38-41], collagen crosslink [42] and degradative enzymes [43,44] to explain the matrix disturbances, and detection of the expression of various cytokines to account for the deregulation of cellular activities [45,46]. Calcified tendinitis exhibited abnormal tendon calcification which is more common in rotator cuffs [47]. Recently, the findings of increased innervations [48] and nociceptive substances [49,50] suggest that the chronic pain of tendinopathy may directly be resulted from the pathological changes. The findings of increased apoptosis [51,52] and acquisition of chondrogenic phenotypes [53] in the injured tendons suggested a disturbance in cell differentiation. Increased proteoglycans with over-sulphation [40] and expression of different versican variant [54] may be related to abnormal chondrogenesis in the affected tendons. In contrast to early observations of a lack of inflammatory cells [11], increased mast cell number was reported in human patellar tendinopathy [55]. Researchers are well aware of the limitation of the clinical samples, which may represent only the end-stage of the pathological processes with unknown duration and onset. Nevertheless, these observations have provided some direct clues to work out the pathogenesis of tendinopathy, and these histopathological characteristics are often used as endpoints in animal models of tendinopathy [56-58]. It should be noted that some of these pathological characteristics are sustained healing responses that failed to repair the initial injury, such as increased cell proliferation and elevated cytokines, which is also implicated in the normal healing process, as shown in the active remodeling sites in healthy tendons [59]. The histopathological features of tendinopathy we observed in animal models must be chronic and cannot be resolved spontaneously as compared to the normal course of tendon healing.

## Genetic predisposition of tendinopathy

The possible genetic predisposition for Achilles tendinopathy has been investigated. It was found that variants within COL5A1 [60], tenascin C [61] and matrix metalloproteinase 3 (MMP3) gene [62] was associated with increased risk of Achilles tendon injuries in general. Since these genes are related to homeostasis of extracellular matrix in tendons, it is suggested that the genetic variants modify the susceptibility of tendons to matrix disturbance observed in tendinopathy.

## Evaluation of interventions to tendinopathy

The observed pathological changes of tendinopathy intuitively provided a lot of insights for the treatments. However, all current treatment methods may not significantly affect the natural history of the disease [63]. Surgical excision was reported to be used on animals over many years, in particular on horses [64]. Surgical excision of pathological tissues [65,66] and percutaneous multiple longitudinal incisions [67,68] were reported to be effective to relieve the symptoms similar to open excision of macroscopic pathologic tendon structures [23]. But ultrasonographic anomalies may still be evident in the healing tissues after surgical excision of pathological tissues; despite the painful symptoms were relieved [69]. Thus the current understanding of the relationship of structural changes and functional impairments is still inadequate to assure the degenerative features as specific "markers" for tendinopathy. Biophysical intervention such as extracorporeal shockwave therapy exhibited significant improvement especially for calcified tendinopathy [70,71]. It suggests that the pathological tissues might be responsive to mechanical stimulation. The observed effects of eccentric exercise for tendinopathy [72,73] also implied that a proper modulation of mechanical environment may exert positive effects on the diseased tendons, such as an increase in peritendinous collagen synthesis [74]. Other biophysical interventions included ultrasound therapy [75-77], pulsed magnetic field therapy [78,79], low level laser therapy [80-82], radiofrequency [83] and acupuncture [84]. These studies claimed that modulation of inflammatory or neuronal components in the pathological tissues may exert beneficial effects. There are also reports on the use of nitric oxide [85], sclerosing agents [86,87], MMP inhibitors [88], bone marrow plasma injection [89], autologous blood injection [90,91] or platelet-rich plasma [92-94] for tendinopathy. Stem cell therapy was tried in horse models [95]. These studies may suggest the involvement of disturbances in cytokines, neovascularization, innervations or cell differentiation in the pathogenesis of tendinopathy.

## Animal models of tendinopathy

The lack of a representative animal model is a major obstacle for tendinopathy research. Recent reviews discussed current animal models used for tendinopathy research [96-98], including cytokine-induced tendon injuries [58,99,100], collagenase-induced injury [56,101-105] and overuse induced injury [57,106-110]. Generally, histopathological characteristics derived from clinical samples are the main criteria for evaluation of tendinopathic changes. Ultrasonographic features were occasionally used in horse models [111], and some studies reported pain-associated behavioral changes associated with the tendon injuries [112,113]. These animal models were established according to different hypotheses of pathogenesis, and aimed at reproducing the clinical signs of tendinopathy as far as possible. The use of cytokines to induce pathological changes implied the key roles of one or several cytokines in the development of the disease; while collagenase injection mimicked the pathological processes from the point when progressive matrix degradation was dominating. These chemically-induced tendinopathy models may reveal different starting points of the pathological process but the causes of increased cytokines or collagenases must be linked with clinically relevant etiological factors. Moreover, the acute induction of degenerative changes in these models cannot reflect the chronic development of the disease. On the other hand, animal models of overuse tendon injuries gained wide acceptance for the demonstration of the relationship between the mechanical overload and the development of histopathological changes [57,109,110] and increase in pro-inflammatory mediators [51,114,115]. Although overuse is sufficient to generate degenerative changes over a longer period of time, this form of injuries can be healed when the overuse training was ceased [116]; while in clinical cases of tendinopathy the symptoms were not improved by rest. Obviously, overuse tendon injury does not equate to tendinopathy. The failed healing response to the injuries caused by mechanical overload of tendons should also be considered in the establishment of animal model of tendinopathy. With respect to the variability in clinical manifestation of tendinopathy, most animal models may only mimic parts of the pathogenesis pathways, or they may only represent one of the possible pathways from the generation of injuries to development of tendinopathy features (Table 1). In summary, it appears that degenerative tendon injuries can be resulted from repetitive strain injuries that exceed the normal thresholds (overuse); while abnormal levels of cytokines and collagenases could be the effectors to mediate this kind of degenerative injuries.

## Cell culture studies of effects of risk factors on tendinopathy

Cell culture studies of tendinopathy included the characterization of abnormal activities in the cells isolated from pathological tissues of tendinopathy [36,44,45,53], and the studies in normal cultured tendon cells in response to potential risk factors such as mechanical strain [117-123] and xenobiotics [124-129]. In cell cultures of tendinopathy tissues, the abnormal cellular activities were persistent during sub-cultures, indicating relatively stable cell phenotypes that are significantly different from tendon fibroblasts derived from healthy tendons [36,45]. On the other hand, numerous studies showed that repetitive mechanical stimulation can affect production of pro-inflammatory mediators [117,118,120-123,130], metalloproteinases [123,131] and matrix syntheses [119] in cultured tendon fibroblasts; while non-tenogenic differentiation of tendon derived stem cells can also be triggered by mechanical stretching [132]. Corticosteroids also induced fibrocartilage phenotype in tendon cells [129], affected matrix synthesis [127], cell viability [126,128] and apoptosis [133]. Fluoroquinolones may also activate metalloproteinases in tendon cells and hence collagenolytic injuries [125]. These observations implied that activation of collagenolysis and erroneous differentiation may weaken the mechanical properties of tendons [134]. Interestingly, non-steroidal anti-inflammatory drugs (NSAIDs) also modulate tendon cell proliferation [124,135], the expression of extracellular matrix components [124] and degradative enzymes [136]. As NSAID is commonly used for sports-related injuries and symptoms, it is possible that anti-inflammatory treatment used for overuse injury may contribute to the development of tendinopathy [137] which is normally diagnosed after NSAID treatment was failed.

## Previous theories of pathogenesis of tendinopathy

Several theories of pathogenesis of tendinopathy have been proposed to explain the development of the histopathological features observed in the clinical samples of tendinopathy. Burry suggested that tendon lesions were not resolved properly and resulted in degenerative changes already in 1978 [138]. However, further elaboration of the idea of "improper resolution of tendon lesion" was not possible due to a lack of experimental evidences at that time. Leadbetter and Khan et al. have suggested that "tendinosis" are degenerative changes resulting from increased demand on tendons with inadequate repair and progressive cell death [139,140]. This model explained the generation of overuse injury, and the reasons for inadequate repair are attributed to adaptive response to tissue overload as elaborated by Kibler and Sorosky et al [141,142]. However, "inadequate repair" as quantitative decrease in healing cells cannot explain the findings of focal hypercellularity, active proliferation and metaplasia in tendinopathy samples. The "apoptosis theory" [143-145] proposed by Murrell also neglect the fact of increased cellularity; but this hypothesis linked up oxidative stress, acquisition of cartilage phenotype and activation of metalloproteinase with the development of degenerative injuries by high dose of cyclic strain. Unfavorable mechanical stimulation as repetitive tensile strain [120], stress-shielding [146], contractile tension overloads [147] or compression [148] was proposed as noxious triggers on tendon cells to induce tendon inflammation or degenerative changes. These theories pointed out that the interactions between tendon cells and their mechanical environment were deterministic for the pathogenesis. On the other hand, Pufe et al. suggested that hypoxia and increased vascular in-growth into tendons may be the causes of tendon weakening and ruptures [149], while Riley provided a neurogenic hypothesis to explain the adaptive responses to mechanical overload by nerve and mast cells unit [150] and Fredberg et al. suggested neurogenic inflammation may be involved in the pathogenesis pathway [151]. These theories were formulated according to the findings of hypervascularity and increased innervations. In summary, the common motifs in these pathogenesis theories include unfavorable mechanical loading, adaptive cellular responses (including tendon, blood vessels and nerves) and the generation of histopathological features. In our opinion, it is possible to unify these ideas as "failed healing", which may be the integral part of various pathological processes that divert various tendon injuries into its different manifestations of tendinopathy. (Table 1)

## A unified theory of pathogenesis of tendinopathy

Based on the information of various lines of investigation of tendinopathy, we can summarize some major points which must be considered in the formulation of the pathogenesis model of tendinopathy:

1. The interactions of tendon injuries and unfavorable mechanical environment would be the starting point of the pathological process. Instead of coining the phrase "adaptive responses of tendon cells", we think that the "adaptive healing responses to tendon injuries" would be a more comprehensive descriptor, which also includes vascular, neural and peri-tendinous reactions at different stages of healing.

2. The normal healing processes are diverted to an abnormal pathway, probably due to unfavorable mechanical environment, disturbances of local inflammatory responses, oxidative stress or pharmacological influences. Therefore, the healing capacity is not only inadequate but also incorrect and deviated from an ideal healing outcome.

3. The primary results of pathology are the progressive collagenolytic injuries co-existing with a failed healing response, thus both degenerative changes and active healing are observed in the pathological tissues.

4. These pathological tissues may aggravate the nociceptive responses by various pathways which are no longer responsive to conventional treatment such as inhibition of prostaglandin synthesis; otherwise the insidious mechanical deterioration without pain may render increased risk of ruptures.

Based on these points, we propose that the pathogenesis of tendinopathy can be perceived as a 3-stages process: injury, failed healing and clinical presentation. The first stage does not involve pathological changes and normal healing response could occur. The second stage is relatively insidious and discriminated from the third stage when clinical presentations are evident, such as ruptures or chronic pain, often resistant to conservative treatments.

**Figure 1:**
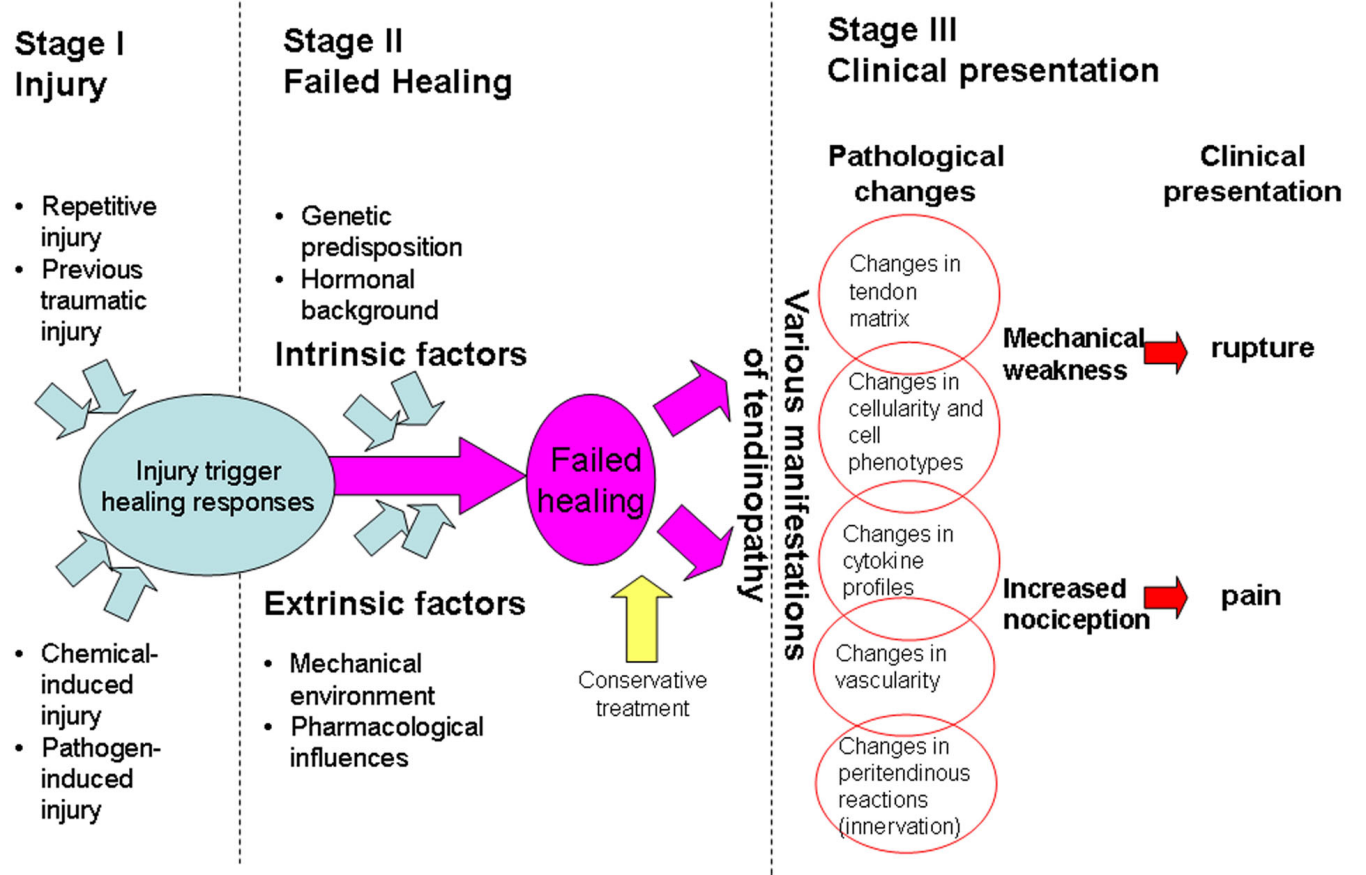
**Failed healing theory for the pathogenesis of tendinopathy**.

Our theory of the pathogenesis of tendinopathy is summarized in Figure 1.

### Stage 1. Injury

In the first stage, initiation of tendinopathy may involve generation of collagenolytic injuries. Overuse can evoke the release of pro-inflammatory mediators [115], which would result in stimulation of metalloproteinases and hence collagenolytic injuries [152,153]. Recent findings showed that the expression of MMP and tissue inhibitor of metalloproteinase (TIMP) in tendon cells were sensitive to mechanical overload or stress-deprivation [131,154]. MMP inhibitor suppressed development of mechanical weakness induced by stress-deprivation [155]. Chemicals such as fluoroquinolone can induce tendon cell death [156], oxidative damages [157] and collagenolysis [125]. A previous traumatic injury that has not been healed may also be susceptible to failed healing [158]. With the case reports of infectious tenosynovitis [159], the involvement of pathogens to generate tendon injuries and inflammation could not be ruled out. Tendon pain and mechanical weakness is not significant at this stage and it is possible for the tendon injury to heal spontaneously.

### Stage 2. Failed healing

In the failed healing stage, healing responses were activated but failed to repair the collagenolytic injuries. The exact causes for failed healing are still obscured. It is speculated that unfavorable mechanical environment, genetic pre-disposition, hormonal background and pharmacological exposures may affect the healing process. Since tendon healing includes many sequential processes such as inflammation, neovascularization, neural modulations [160], recruitment of healing cells, proliferation, apoptosis [161], matrix synthesis, tenogenic differentiation and matrix remodeling; disturbances occurred at different stages of healing may lead to different combinations of histopathological changes as what we observed in the clinical samples of tendinopathy (Table 1). Inflammatory responses are presumably elicited as the initial stage of tissue repair, but it may not be properly resolved under hostile mechanical environment or pharmacological intervention such as NSAIDs, resulting in elevated pro-inflammatory cytokines and a lack of ordinary inflammatory infiltration in the diseased tissues. Depending on the anatomical variations of the affected tendons, peritendinous reactions may be resulted as restrictive fibrosis, increased innervations and vascular in-growth from para-tenon structures may also take part in healing process [162]. The sustained activation of tendon progenitor cells with unfavorable micro-milieu for tendogenic differentiation may prone to erroneous differentiation into fibrochondrogenic [163] or calcifying phenotypes [104], which are normally confined to the regions of bone-tendon junctions. Tendon pain becomes significant and conservative treatments such as NSAIDs are prescribed to the patients, which may further modify the pathways of the failed healing.

### Stage 3. Clinical presentation

In the third stage, symptomatic tendinopathy is diagnosed as longstanding, activity-related pain with characteristic medical images; while spontaneous ruptures are resulted from mechanical weakness under normal activities in cases of asymptomatic tendinopathy. The consequences of failed healing to collagenolytic injuries involve significant changes in extracellular matrix, which are then visible under ultrasound or MRI. In symptomatic cases, inflammatory pain may be involved and controlled during the injury and failed healing stages, but the pain mechanism may gradually shift to non-phlogistic ones such as agitation to peritendinous nerves by nociceptive substances or swelling, rendering the resistance to common anti-inflammatory treatments. Though mechanical weakness may be involved in asymptomatic cases [164], lower activities due to pain may reduce risk of ruptures. In asymptomatic cases, the matrix disturbance resulted from failed healing may not activate nociceptive response. The insidious deterioration in mechanical properties of the affected tendons may lead to ruptures. Owing to different combinations of etiological factors, temporal and spatial variations on the failed healing, the clinical manifestations of tendinopathy may exhibit high variability.

## Explicability of the theory

With this theory for the pathogenesis of tendinopathy, we can explain the process of the generation of the pathological features of tendinopathy we observed in the clinical samples. The theory is in accordance with most of the evidences derived from tendinopathy studies. For example, overuse is a major etiological factor but there are tendinopathy patients without obvious history of repetitive injuries. It is possible that non-overuse tendon injuries may also be exposed to risk factors for failed healing and entered to the third stage of tendinopathy. Overuse induces collagenolytic tendon injuries and it also imposes repetitive mechanical strain which may be unfavorable for normal healing. Stress-deprivation also induces MMP expression and whether over- or under-stimulation is still an active debate [165]. It is possible that tenocyte is responsive to both over- and under-stimulation, both tensile and compressive loading. Because the cellular responses of healing tendon cells change in different stages of tendon healing [166], we speculate that the cell responsiveness to mechanical loading may not be constant during tendon healing and failed healing may be resulted from a mismatch of healing stages and the mechanical environment. Our theory can also explain why animal models of collagenase-induced injuries can reproduce the histopathological characteristics and functional impairment similar to tendinopathy; despite the generation of collagenolytic injuries in these models are completely different from the insidious onset of tendinopathy. By proposing a process of failed healing to translate tendon injuries into tendinopathy, other extrinsic and intrinsic factors would probably enter the play at this stage, such as genetic predisposition, age [167], xenobiotics (NSAIDs and corticosteroids) and mechanical loading on the tendons. For example, differential tensile forces acting on patellar tendon [168] may impose varying loading on tendon cells in different regions, it may explain why posterior proximal patellar tendon is pathological in patellar tendinopathy. Peritendinous structures may be disturbed to different extents in the healing response to tendon injuries, which may lead to different manifestations of "paratenonitis" or tendon adhesion. Investigations of how these factors affect tendon healing could help to further elucidate the mechanism of failed healing. The recent discovery of tendon-derived stem cells and characterization of pathological tissues of tendinopathy have provided evidences to support the ideas of erroneous cell differentiation that contribute to failed tendon healing. According to this theory of pathogenesis, we shall have a theoretical framework to develop a more representative animal model of tendinopathy for further study and verification. New ideas for treatments of tendinopathy may be inspired based on this theory, for example, a treatment which could override on the failed healing tissues and restart the healing process.

### Missing links and limitations

As compared to previous theories of pathogenesis for tendinopathy which described a viscous cycle of inadequate repair and increased susceptibility of further injuries, this new theory attempts to describe the "vicious cycle" as an interaction between the vulnerability of the healing process to noxious mechanical and biochemical environments. Thus we can investigate the missing links as predicted in the theory, for example, the impact of mechanical stimulation on the cell differentiation of healing tendons cells, and the disturbances in cytokines triggered by re-injury on healing tendons. However, there are still some limitations in the current pathogenesis theory. Firstly, tendinopathies in different tendons exhibited specific patterns of affected regions and different forms of matrix disturbance, which may be presumably accounted by variations in local mechanical environment and vascular supplies; but it is difficult to explain for these variations at the present stage. Secondly, the interplay among innervations, increased nociception and tendon healing is unknown. It is still a black box for the mechanism of increased nociception by failed tendon healing. The factors which govern the development of chronic pain in tendinopathic tendons remain obscure. Finally, the interactions between healing tendons and the peritendinous tissues are seldom investigated and it is difficult to evaluate the potential involvement of peritendinous tissues in the development of tendinopathy.

## Conclusions

In summary, we propose a unified theory for pathogenesis of tendinopathy which explain most of the available experimental data about tendinopathy. It is a just a start to probe into the nature of the pathology and we certainly wait for new findings and challenges to this theory until we finally find the truth.

## Abbreviations

MMP3: Matrix metalloproteinase 3; MRI: Magnetic resonance imaging; NSAID: Non-steroidal anti-inflammatory drug; TIMP: Tissue inhibitor of metalloproteinase

## Competing interests

The authors declare that they have no competing interests.

## Authors' contributions

SCF, RC and KMC planned and drafted the manuscript. YCC managed the references and assisted in drafting. PPYL approved the final version. All authors read and approved the final manuscript.
